# Genomic characterization and tissue tropism variations of two porcine delta coronavirus strains isolated in China

**DOI:** 10.3389/fcimb.2024.1507277

**Published:** 2024-12-02

**Authors:** Guangli Hu, Yihui Huang, Zexin Chen, Rui Geng, Zhiqing Zhao, Ouyang Peng, Chuangchao Zou, Hanqin Shen, Yongchang Cao, Hao Zhang

**Affiliations:** ^1^ State Key Laboratory of Biocontrol, School of Life Sciences, Sun Yat-sen University, Guangzhou, China; ^2^ Guangdong Enterprise Key Laboratory for Animal Health and Environmental Control, Wen’s Foodstuff Group Co. Ltd, Yunfu, China

**Keywords:** porcine delta coronavirus, PDCoV, immunogenicity, tissue tropism, isolation and identification

## Abstract

The porcine delta coronavirus (PDCoV) is a member of the Delta coronavirus genus, which can lead to diarrhea, vomiting, and mortality in piglets. First detected in Hong Kong in 2012, PDCoV has since spread globally. In January 2024, two strains, CHN-ANHZ-2024 and CHN-JSSQ-2024, were isolated from diarrheal piglets in Anhui and Jiangsu provinces. Immunofluorescence assays, electron microscopy, and genome sequencing were performed. Genome analysis revealed that both PDCoV strains belonged to the Chinese lineage, exhibiting amino acid mutations in the S1 region compared to other strains within the lineage. Amino acid mutation at position 530L is uniquely associated with the Thai strain. Notably, CHN-JSSQ-2024 was identified as a recombinant strain of DH1 and CHN-AHHN-2024, with the recombination occurring in the S2 subunit. CHN-ANHZ-2024 caused severe diarrhea with an 80% mortality rate, whereas CHN-JSSQ-2024 resulted in mild diarrhea without mortality. Viral load analysis showed CHN-ANHZ-2024 primarily infecting the brain and kidneys, while CHN-JSSQ-2024 targeted the lungs, revealing notable differences in tissue tropism. We designed the RNA scope Probe-PDCoV-N to visualize viral RNA in the positively detected organs, viral RNA was detected in the brain, cerebellum, kidneys, and lungs of the infected piglets. This study highlights significant differences in the pathogenicity and organ tropism of two PDCoV strains. The CHN-ANHZ-2024 strain caused severe diarrhea and high mortality in piglets, while the CHN-JSSQ-2024 strain exhibited much milder symptoms. Additionally, the study elucidated notable differences in organ tropism between the strains, offering valuable insights into the epidemiological characteristics and pathogenic mechanisms of PDCoV. These findings provide a foundation for the development of targeted prevention and treatment strategies tailored to specific strains in the future.

## Introduction

PDCoV is an enveloped, positive-sense single-stranded RNA virus with a genome size of 25 kb, classified under the Delta coronavirus genus in the order Nidovirales (Zhang, 2016). The genome encodes two large polyproteins, ORF1a and ORF1b, four structural proteins, including spike (S), envelope (E), and membrane (M), as well as two accessory proteins, NS6 and NS7 (Lee and Lee, 2014; Fang et al., 2017; Chen et al., 2020).

PDCoV can cause severe diarrhea, vomiting, dehydration, and mortality in piglets (Chen et al., 2015; Ma et al., 2015; McCluskey et al., 2016; Zhang et al., 2019). It was initially reported in pigs in Hong Kong in 2012 (Woo et al., 2012), with subsequent cases emerging in various countries and regions worldwide, such as the United States (Wang L. et al., 2014), South Korea (Lee and Lee, 2014), Thailand (Janetanakit et al., 2016), Japan (Suzuki et al., 2018), and China (Dong et al., 2015). The widespread prevalence of PDCoV among global pig populations has resulted in significant economic losses in the pig farming industry. Notably, PDCoV can cause respiratory infections in pigs and can be transmitted via the respiratory route (Woo et al., 2017). It has also been detected in multiple organs of infected piglets, including the heart, liver, spleen, lungs, kidneys, and stomach (Jung et al., 2015). Additionally, PDCoV is capable of infecting cattle, mice, and poultry (Jung et al., 2017; Li et al., 2018; Liang et al., 2019). Furthermore, PDCoV has been detected in plasma samples from three Haitian children suffering from acute undifferentiated febrile illness (Lednicky et al., 2021). The broad host range and diverse tissue tropism of PDCoV highlight its potential for cross-species transmission, posing an emerging threat to animal and possibly human health.

Although PDCoV has been reported in various countries, there is limited information on the regional genetic diversity and tissue tropism of Chinese strains. This study aims to address this gap by characterizing two novel strains from Anhui and Jiangsu provinces. In this study, we isolated two strains of PDCoV, designated CHN-ANHZ-2024 and CHN-JSSQ-2024, from LLC-PK1 cells in Anhui and Jiangsu provinces of China. The complete genomes were sequenced, confirming that both PDCoV strains belong to the Chinese lineage, with CHN-JSSQ-2024 identified as a recombinant strain of DH1 and CHN-AHHN-2024. We assessed their pathogenicity through clinical evaluations, quantification of viral shedding, histological examinations, and immunohistochemical assays. Additionally, we designed an RNA scope probe, PDCoV-N, to visualize the distribution of the virus in intestinal tissues and organs. The results demonstrated that the PDCoV CHN-ANHZ-2024 strain caused severe diarrhea and an 80% mortality rate in piglets, whereas CHN-JSSQ-2024 resulted in only mild diarrhea without any associated mortality. In addition to the viral distribution detected in the intestine, CHN-ANHZ-2024 was also found to infect the brain and kidneys, while CHN-JSSQ-2024 primarily targeted the lungs. This observation highlights the differences in tissue tropism between the two strains. This study seeks to (1) characterize the genomic differences between CHN-ANHZ-2024 and CHN-JSSQ-2024 (2), compare their pathogenicity in piglets, and (3) investigate differences in tissue tropism.

## Materials and methods

### Clinical sample collection

In January 2024, clinical samples were collected from two pig farms in Anhui and Jiangsu provinces experiencing piglet diarrhea. From the Anhui farm, 158 samples were obtained, including 9 piglet intestinal tissue samples and 149 fecal samples. Meanwhile, 24 samples were collected from the Jiangsu farm, comprising 1 intestinal tissue sample and 23 fecal samples. The tissue samples were placed in 2 mL centrifuge tubes containing 1 mL of DMEM medium, homogenized, subjected to freeze-thaw cycles, and then centrifuged at 12,000 rpm for 5 minutes. Supernatants were stored at -80°C for further analysis. Fecal samples were diluted 1:100 in phosphate-buffered saline (PBS) and used for RNA extraction with the MagaBio Plus Virus DNA/RNA Purification Kit II (MagaBio, China), following the manufacturer’s protocol. Porcine epidemic diarrhea virus (PEDV), PDCoV, transmissible gastroenteritis virus (TGEV), swine acute diarrhea syndrome coronavirus (SADS-CoV), and porcine rotavirus (PoRV) were detected using the Hifair^®^ V C58P2 Multiplex One Step RT-qPCR Probe Kit (Yeasen Biotechnology, China).PEDV sense: 5-GAATTCCCAAGGGCGAAAAT-3′: antisense:5′-TTTTCGACAAATTCCGCATCT-3′: probe:CGTAGCAGCTTGCTTCGGACCCA),PDCoV sense: 5′-ATCGACCACATGGCTCCAA-3′: antisense:5′-CAGCTCTTGCCCATGTAGCTT-3′: probe:CACACCAGTCGTTAAGCATGGCAAGCT,TGEV sense: 5′-CCACCACAGAATCTAGTTTGACTTGC-3′: antisense:5′-ACCGCATCTGCAAACCATTGTAGG-3′: probe:GTGAGTGCAGGTTAAACCATAAGTTCCCT,SADS-CoV sense: 5′-CTGACTGTTGTTGAGGTTAC-3′: antisense:5′-TCTGCCAAAGCTTGTTTAAC-3′: probe:TCACAGTCTCGTTCTCGCAATCA,PoRV sense: 5′-GGACTTACATTACGAATTGAATCTG-3′: antisense:5′-TTGCCAAYAAAGTTTCRGAAGC-3′: probe:AGTTTGTGAATCTGTGCTTGCGGA.The one-step RT-PCR was conducted in a 20 μL reaction volume, containing 2 μg of RNA, 10 μL of 2× Hifair^®^ V C58P2 MP Buffer, 0.8 μL of Hifair^®^ V C58P2 Enzyme Mix, 0.4 μmol/L of each gene-specific primer, and 0.2 μmol/L of each gene-specific probe. RNase-free H₂O was added to bring the volume up to 20 μL. The thermal cycling conditions were as follows: 50°C for 20 min, 95°C for 5 min, followed by 40 cycles of 95°C for 15 s and 60°C for 30 s.

### Isolation and identification of PDCoV in LLC-PK cells

LLC-PK cells were obtained from Guangdong Wen’s Foodstuffs Group Co. Ltd (Guangdong, China) and cultured in DMEM medium (Hyclone, USA) supplemented with 10% fetal bovine serum (FBS) (BOVOGEN, Australia). PDCoV amplification was performed in DMEM supplemented with 8 µg/mL trypsin (Gibco, USA) in a 5% CO₂ incubator. After centrifugation of the homogenized positive tissue at 10,000 rpm for 10 minutes, the supernatant was mixed with DMEM at ratios of 1:10, 1:20, and 1:50, and then filtered through a 0.22 μm filter for sterilization. A volume of 500 µL of the mixture was added to monolayer LLC-PK cells in a six-well plate and incubated for 1.5 hours, after which the supernatant was discarded. Subsequently, 2 mL of DMEM containing 2% FBS and 1% dual-specific antibodies was added, and the cells were incubated at 37°C in a 5% CO₂ incubator for 48 hours to observe for cytopathic effects (CPE). If no CPE was observed, the cells underwent three freeze-thaw cycles at −80°C. The cells were then collected and passaged until approximately 80% exhibited CPE. During passage, positive control wells inoculated with PDCoV and negative control wells without virus inoculation were included.

### The infectious virus titration was conducted using the TCID50 assay

LLC-PK cells were seeded in 96-well plates, cultured overnight, and subsequently washed twice with maintenance medium. A total of 100 μL of 10-fold serially diluted PDCoV was inoculated into eight wells per dilution and incubated at 37°C in a 5% CO₂ environment. The cytopathic effect (CPE) was monitored for 5 to 7 days, and virus titers were calculated using the Reed-Muench method (Reed and Muench, 1938) expressed as TCID50/mL.

### Immunofluorescence assay

LLC-PK cells in 6-well cell culture plates were either mock-infected or infected with porcine epidemic diarrhea virus (PEDV) at a multiplicity of infection (MOI) of 0.1. At 24 hours post-infection, the cells were fixed with 4% paraformaldehyde at 4°C for 30 minutes and then permeabilized with 0.25% Triton X-100 (Solarbio, China) for 10 minutes at room temperature. After washing the plates three times with phosphate-buffered saline (PBS) (Gibco™, USA), the cells were blocked with 5% bovine serum albumin (BSA; Solarbio, China) at room temperature for 1 hour. Mouse anti-PDCoV spike (S) protein (preserved in this laboratory) and Alexa Fluor^®^ 488-conjugated goat anti-mouse IgG (Abcam, UK) were used as primary and secondary antibodies, respectively. The cell nuclei were stained with 4′,6-diamidino-2-phenylindole (DAPI; Vectorlabs, USA) for 5 minutes at room temperature. After washing with PBS, the stained cells were observed using a fluorescence microscope (Olympus, Japan).

### RNA extraction, library construction, and sequencing

RNA was extracted from samples of clarified infected cell lysate or purified virus using TRIzol and prepared for next-generation sequencing. Briefly, reverse transcription was performed using random hexamers. Subsequent DNase treatment and cleanup were followed by second-strand synthesis before library preparation, which utilized Nextera XT reagents (Illumina) and sequencing on the NovaSeq 6000 platform (Illumina). Although originally described as a consensus-level sequencing methodology, the depth of coverage allowed for deep sequencing analysis. Bioinformatics analysis of the data was completed using the previously described pipeline.

### Genome sequencing, assembly and annotation

Raw reads were filtered and trimmed using fastp (https://github.com/OpenGene/fastp) to remove sequencing adapters and low-quality reads, including those with a quality score of <Q20. Ribosomal RNA and host reads were subtracted through read mapping using the BBMAP program. *De novo* genome assembly was performed with SPAdes v3.13.0 (Nurk et al., 2013). The extracted assembled scaffolds were limited to a minimum contig length of 100 bases, with the best BLAST hits obtained from the NCBI nt database.

### Phylogenetic analysis of the spike and gene and full-length genome sequence

The sequence fragments were assembled and analyzed using DNAStar 7.0 and BioEdit software, respectively. Complete sequences of the S gene and full-length genome reference sequences obtained from GenBank were used for sequence alignment and phylogenetic analyses. Phylogenetic trees were constructed using the neighbor-joining method in MEGA version 6, with bootstrap values calculated for each node from 1,000 replicates. All tree figures were produced using MEGA 4.0 software.

### Pathogenicity of the CHN-ANHZ-2024 and CHN-JSSQ-2024strain in piglets

All animals were obtained from a conventional breeding farm with a good health record and no prior history of Porcine Delta Coronavirus (PDCoV) outbreaks; they were confirmed negative for any porcine enteric viruses. Fifteen 3-day-old piglets were randomly assigned to three groups of five and housed in separate rooms. The piglets were fed commercial fresh milk five times daily and had unrestricted access to distilled water throughout the experiment. Before the challenge, rectal swabs confirmed that the piglets were negative for major porcine enteric viruses PEDV, TGEV, PDCoV, SADS CoV, and PoRV via RT-qPCR. The two groups were orally inoculated with either 1 mL of 1 × 10^6^ TCID50 of CHN-ANHZ-2024 or 1 mL of 1 × 10^6^ TCID50 of CHN-JSSQ-2024, the mock infection group was orally inoculated with 1 mL of DMEM. Following inoculation, the piglets were monitored daily for clinical symptoms such as depression, slow movement, diarrhea, vomiting, and anorexia. Rectal swabs were collected daily to monitor fecal viral RNA shedding using quantitative real-time RT-PCR targeting the PDCoV M gene. All surviving pigs were euthanized at the end of the experiment (5 days post-inoculation [dpi]).The clinical significance score (CSS) was assessed using the following criteria for diarrhea severity based on fecal consistency at 5 dpi: 0 = normal with no diarrhea; 1 = soft stool; 2 = mildly fluidic feces; 3 = moderately mucous-to-watery diarrhea; and 4 = severe watery and projectile diarrhea. To evaluate differences in pathogenicity among PDCoV strains during the acute phase of infection, experiments were performed on three piglets per group. Intestinal damage and viral load in intestinal tissues of piglets infected with different strains were assessed. All piglets were euthanized at 3 dpi, and samples from the small intestine, heart, lung, spleen, liver, kidney, brain, and cerebellum were collected and fixed in 4% paraformaldehyde for 48 hours. Fixed samples were subjected to hematoxylin and eosin (H&E) staining or immunohistochemistry (IHC) with a PDCoV N-specific monoclonal antibody.

### RNA *in situ* hybridization

The RNAscope *in situ* hybridization (ISH) method for formalin-fixed paraffin-embedded (FFPE) tissues has been previously described (Wang H. et al., 2014). To detect the distribution of Porcine Delta Coronavirus (PDCoV) nucleic acids in intestinal and organ tracts, ISH was performed on FFPE intestinal tissues using the RNAscope^®^ Multiplex Fluorescent Reagent Kit v2 (Cat. No. 510991, Advanced Cell Diagnostics, Inc, USA), according to the manufacturer’s instructions. Briefly, 20 double Z probe pairs specifically targeting the region coding for the PDCoV nucleocapsid protein were designed and synthesized by ACD. Each probe forms a 28-base hybridization with the targeted RNA, allowing for the preamplifier to contain 20 binding sites for the amplifier. Probes were fluorescently labeled with cyanine 3 for direct visualization under a fluorescence microscope. For tissue processing, the FFPE intestinal tissues were cut into 5-μm-thick sections, and tissue sections on slides were baked in a dry oven at 60°C for 1 hour before being deparaffinized in xylene and dehydrated through an ethanol series. Finally, slides were placed on absorbent paper with the section face up and air-dried for 5 minutes at room temperature (RT). Following this, the sections were treated with several drops of RNAscope Hydrogen Peroxide for 10 minutes at RT and then washed in distilled water 3–5 times. Afterwards, tissue sections were incubated in citrate buffer (10 nmol/L, pH 6) maintained at boiling temperature for 15 minutes. Immediately, the sections were rinsed in deionized water and transferred into 100% alcohol for 3 minutes before drying the slides in a 60°C incubator. Next, tissue sections were treated with 10 μg/mL protease at 40°C for 30 minutes in a HybEZ hybridization oven (ACD) and washed 3–5 times in distilled water. Then, the tissue sections were incubated at 40°C in the following order: target probes for 3 hours; preamplifier for 30 minutes; amplifier for 15 minutes; and label probe for 15 min. For each hybridization step, tissues were rinsed with wash buffer 3 times at RT after treatment. Hybridization signals were detected by TSA^®^ Plus Cyanine 3 (NEL744E001KT, PerkinElmer). At last, the sections on glass slides were counterstained with 4′,6-diamidino-2-phenylindole (DAPI). The images were captured by a Nikon A1 confocal microscope and processed by image J.

## Results

### Clinical detection of PDCoV

**Figure 1:**
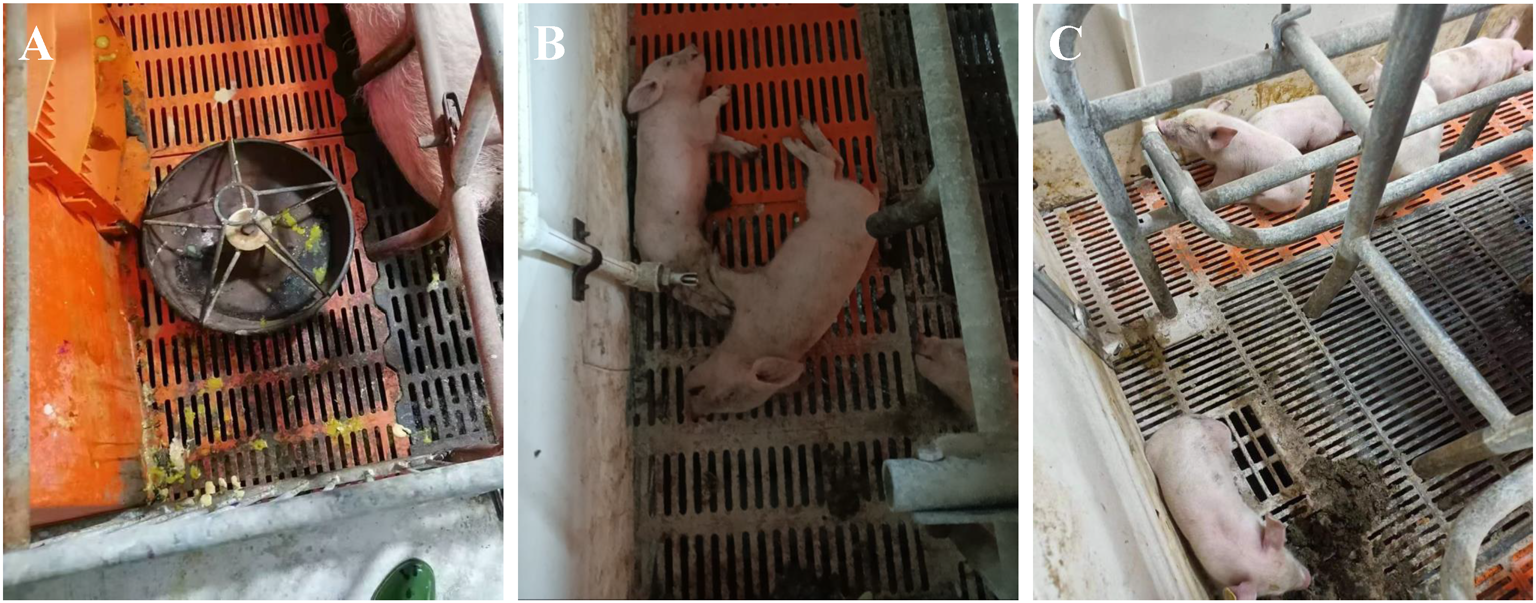
Clinical yymptoms of PDCoV in Pig Farms. **(A)** Piglets present with watery diarrhea. **(B)** Piglets succumb to severe diarrhea. **(C)** Piglets exhibit rough hair and lethargy. Panels A and B represent the clinical manifestations observed in piglets at a farm in Anhui. Panel C illustrates the clinical manifestation observed in piglets at a farm in Jiangsu.

In January 2024, pig farms in Anhui and Jiangsu provinces of China reported cases of diarrhea in piglets. Clinical samples were tested for PEDV, PDCoV, TGEV, SADS-CoV, and PoRV by RT-qPCR. In the Anhui farm, all 33 fecal or intestinal samples were positive for PDCoV (100%), while tests for PEDV, TGEV, SADS-CoV, and PoRV were negative. In this farm, 24 piglets died (73%), subsequently leading to the infection of other piglets, which caused severe diarrhea and death in 134 piglets (**Figures 1A, B**). On the Jiangsu farm, 13 out of 24 fecal or intestinal samples (54%) tested positive for PDCoV, while other diarrhea-related pathogens were negative. The piglets on this farm exhibited only mild diarrhea and vomiting (**Figure 1C**), with no deaths recorded.

### Virus isolation and identification

**Figure 2:**
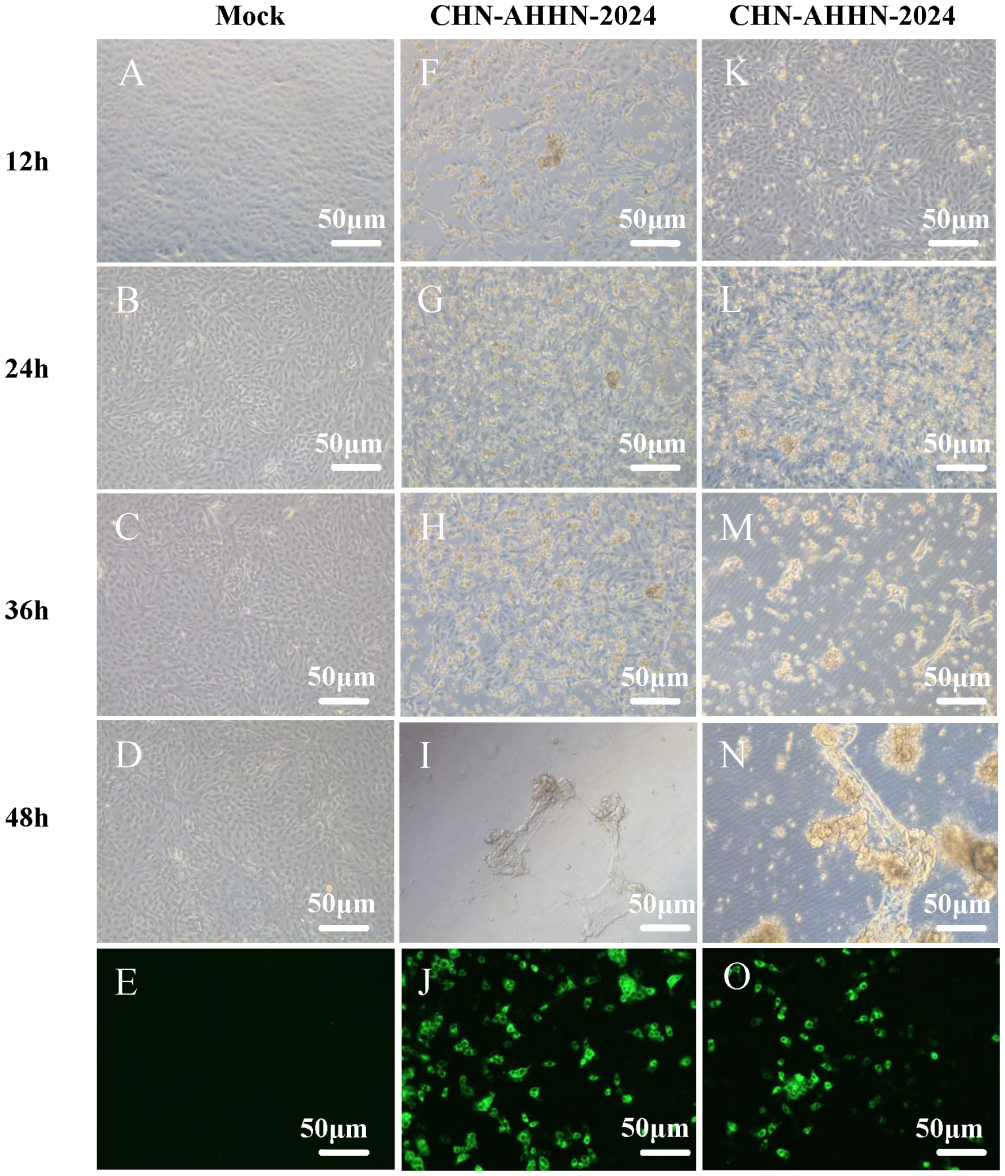
Cytopathic Effects (CPE) and Immunofluorescence Analysis (IFA) Staining in LLC-PK Cells Inoculated with PDCoV. **(A–D)** Mock-inoculated LLC-PK cell culture displays normal cells. **(G–J)** PDCoV-CHN-ANHZ-2024-inoculated LLC-PK cells exhibit rounded and clustered morphology between 12 and 48 hours post-infection. **(O–R)** PDCoV-CHN-JSSQ-2024-inoculated LLC-PK cells exhibit rounded and clustered morphology between 12 and 48hours post-infection. Detection of PDCoV in LLC-PK cells was performed by IF staining using mouse anti-PDCoV antiserum. **(E)** IF staining of mock-incubated LLC-PK cells, with LLC-PK cells fixed at 24 hours post-infection. **(L)** IF staining of PDCoV-CHN-ANHZ-2024-infected LLC-PK cells; **(M)** IF staining of PDCoV-CHN-JSSQ-2024-infected LLC-PK cells.

**Figure 3:**
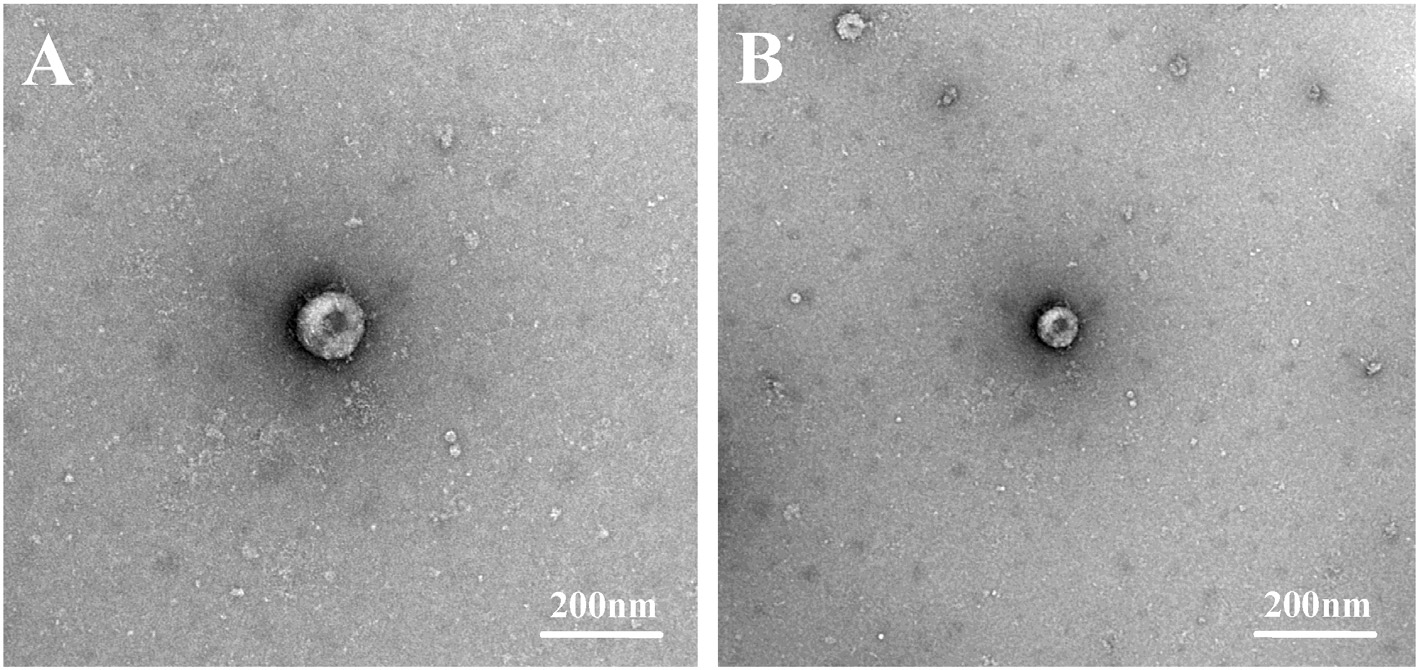
Electron Micrographs of PDCoV-CHN-JSSQ-2024. **(A)** and PDCoV-CHN-ANHZ-2024 **(B)** Inoculated into LLC-PK Cells. Crown-shaped spikes are visible. The samples were subjected to negative staining with 3% phosphotungstic acid.

After grinding, centrifuging, and filtering intestinal tissues from PDCoV-positive piglets, the supernatant of two PDCoV-positive samples was diluted and used for the inoculation of LLC-PK cells, which were then passaged. Two PDCoV strains were successfully isolated, designated PDCoV CHN-ANHZ-2024 and CHN-JSSQ-2024. In LLC-PK cells, the cytopathic effects (CPE) of PDCoV manifested as cell aggregation and rounding, resulting in cell death within 24–36 hours post-infection (**Figures 2F–N**). Uninfected control cells (**Figures 2A–D**) remained flat and adherent to the culture plate. At 24 hours post-infection, the immunofluorescence assay (IFA) detected the PDCoV N protein in both CHN-ANHZ-2024 and CHN-JSSQ-2024. As shown in **Figure 2E**, no fluorescence was observed in uninfected control cells, while green fluorescence, corresponding to viral presence, was observed in cells infected with CHN-ANHZ-2024 and CHN-JSSQ-2024 (**Figure 2J**) and CHN-JSSQ-2024 (**Figure 2O**). Electron microscopy (EM) revealed the morphology and size of CHN-ANHZ-2024 and CHN-JSSQ-2024 virus particles purified from infected LLC-PK cells. Using negative staining, typical coronavirus-like virions with spike projections and diameters of 80–150 nm were observed under EM (**Figures 3A, B**). These results confirm the successful isolation of two PDCoV strains from the samples.

### Phylogenetic analysis and genetic divergence of the PDCoV S gene

**Figure 4:**
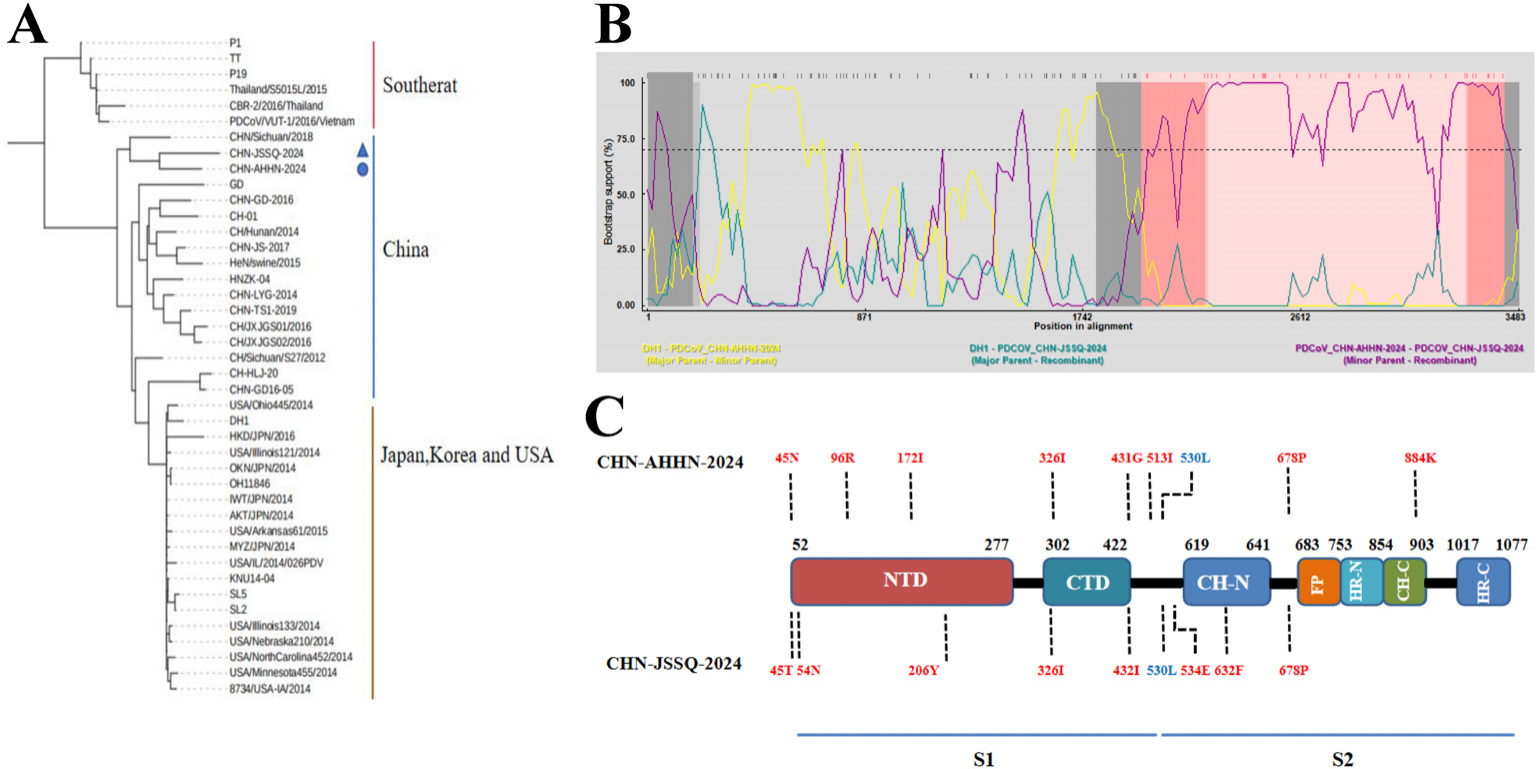
Phylogenetic analysis of PDCoV-CHN-JSSQ-2024 and PDCoV-CHN-ANHZ-2024. **(A)** Phylogenetic Analysis of the PDCoV S Protein Genomic Sequence Using the Neighbour-Joining Algorithm and 1,000 Bootstrap Replications in a Heuristic Search with 40 PDCoV Reference Strains. **(B)** Recombination Analysis by Screening Multiple Sequence Alignments of the PDCoV S Gene with the Recombination Detection Program (RDP). The pairwise identities of the potential recombinant CHN-JSSQ-2024, the major parent DH1, and the minor parent CHN-AHHN-2024 determine the potential recombinant region with a 95% confidence interval. **(C)** Compared to the Chinese Strains of PDCoV, Mutations in the S Gene Amino Acids of CHN-JSSQ-2024 and AHHN-2024 are Highlighted in Red, with Blue Indicating the Mutation Sites that are the Same as those in the Thai Strain.

Sequence analysis revealed that CHN-ANHZ-2024 and CHN-JSSQ-2024 are part of the Chinese lineage of PDCoV strains (**Figure 4A**). Compared with other reported Chinese PDCoV strains, the S genes of CHN-ANHZ-2024 and CHN-JSSQ-2024 harbor several novel mutations. Specifically, nine nucleotide substitutions were identified within the S genes of both strains (**Figure 4C**). To investigate the genetic characteristics of CHN-ANHZ-2024 and CHN-JSSQ-2024, these strains, along with other reference strains, were analyzed using the Recombination Detection Program version 4.9.4 (RDP4) for evidence of genetic recombination. As shown in **Figure 4A**, no evidence of recombination was detected in CHN-ANHZ-2024. In contrast, CHN-JSSQ-2024 emerged as a result of recombination involving strain DH1 as the major parent and CHN-ANHZ-2024 as the minor parent, with recombination breakpoints mapped to positions 1971 (start) and 3421 (end) (**Figure 4B**). These findings suggest that CHN-JSSQ-2024 resulted from a natural recombination event between the DH1 and CHN-ANHZ-2024 strains.

### CHN-ANHZ-2024 exhibits high pathogenicity in 3-day-old piglets, whereas CHN-JSSQ-2024 induces only mild clinical symptoms in infected piglets

**Figure 5:**
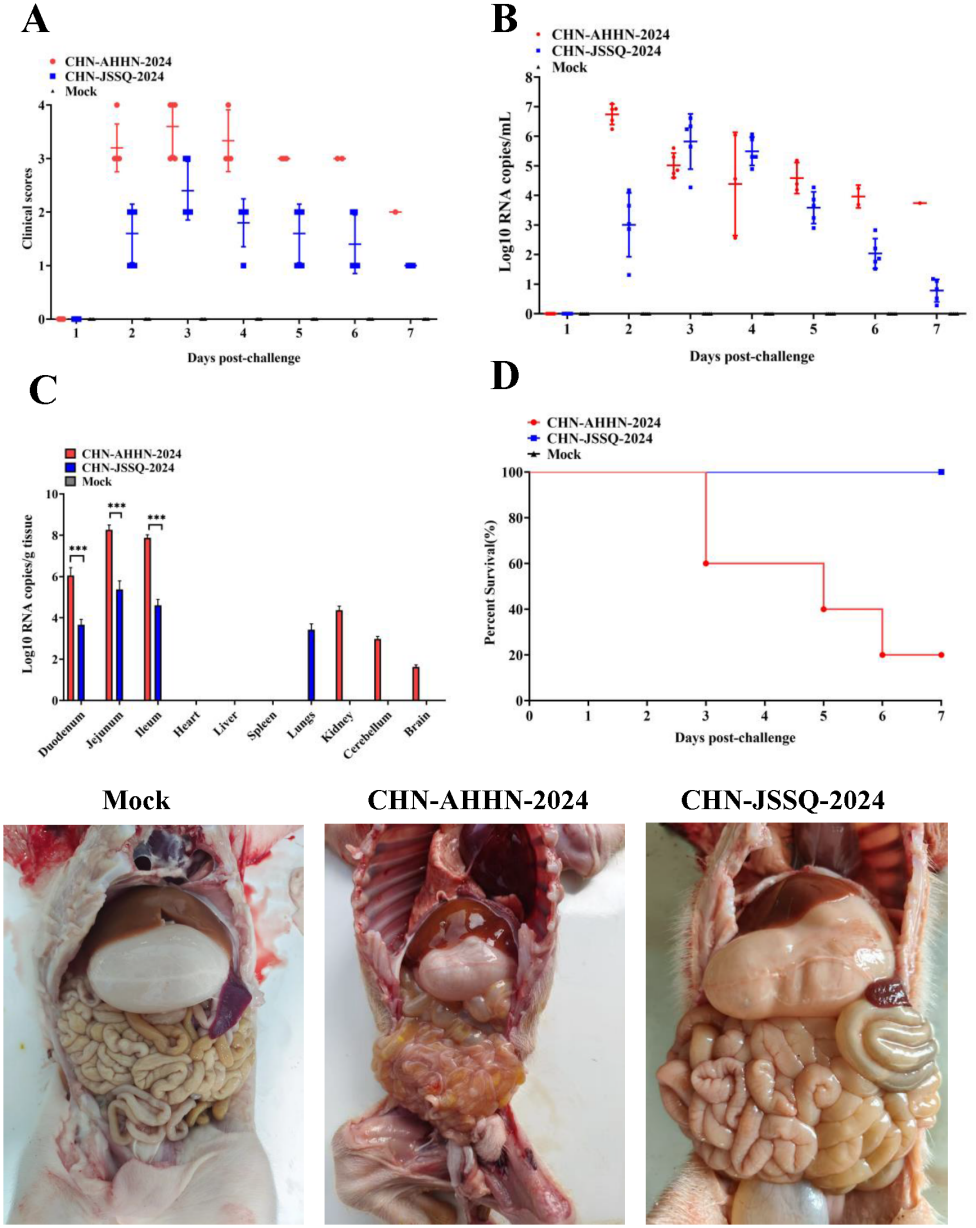
PDCoV Infection in Pigs Leads to Fecal Virus Shedding, Clinical Scoring, Virus Distribution, and Survival Rate. **(A)** Clinical Scoring in 3-Day-Old Pigs Challenged with PDCoV Strain CHN-AHHN-2024 and CHN-JSSQ-2024. **(B)** Fecal Virus Shedding in 3-Day-Old Pigs Challenged with PDCoV Strain CHN-AHHN-2024 and CHN-JSSQ-2024. **(C)** Viral RNA Distribution in 3-Day-Old Pigs Challenged with PDCoV Strain CHN-AHHN-2024 and CHN-JSSQ-2024. **(D)** Survival Rate in 3-Day-Old Pigs Challenged with PDCoV Strain CHN-AHHN-2024 and CHN-JSSQ-2024. Significant differences between groups are marked by asterisks (***P < 0.001).

To evaluate the pathogenicity of the isolated PDCoV strains CHN-ANHZ-2024 and CHN-JSSQ-2024, 15 piglets were randomly assigned to three groups of five and housed separately. The piglets were orally inoculated with either CHN-ANHZ-2024 or CHN-JSSQ-2024 at 1 mL of 1×10^6^ TCID50 per piglet, while the control group orally inoculated with 1 mL of DMEM. Piglets infected with CHN-ANHZ-2024 manifested acute watery diarrhea, vomiting, and severe dehydration (**Figure 5A**). In contrast, those infected with CHN-JSSQ-2024 displayed only mild diarrhea and slight dehydration (**Figure 5A**). By day 7, the survival rate for CHN-ANHZ-2024-infected piglets was 20%, whereas the survival rate of infected with CHN-JSSQ-2024 was 100% (**Figure 5D**). Viral RNA was measured by qRT-PCR in fecal swabs collected from day 1 to day 7, with CHN-ANHZ-2024 showing consistently higher viral loads than CHN-JSSQ-2024. No viral RNA was detected in the control group (**Figure 5B**). Post-infection, PDCoV were found in the duodenum, jejunum, and ileum of the infected piglets. Notably, CHN-ANHZ-2024 was also present in the brain, cerebellum, and kidneys, whereas CHN-JSSQ-2024 was localized primarily in the lungs (**Figure 5C**). These findings suggest that CHN-ANHZ-2024 is highly pathogenic, causing severe watery diarrhea and high mortality, while CHN-JSSQ-2024 induces only mild symptoms without mortality. Additionally, the two strains exhibit distinct organ tropism: CHN-ANHZ-2024 primarily targets the brain, cerebellum, and kidneys, while CHN-JSSQ-2024 predominantly infects the lungs.

### Histopathological and immunohistochemical stainingy in 3-day-old piglets Infected with PDCoV CHN-ANHZ-2024 and CHN-JSSQ-2024

**Figure 6:**
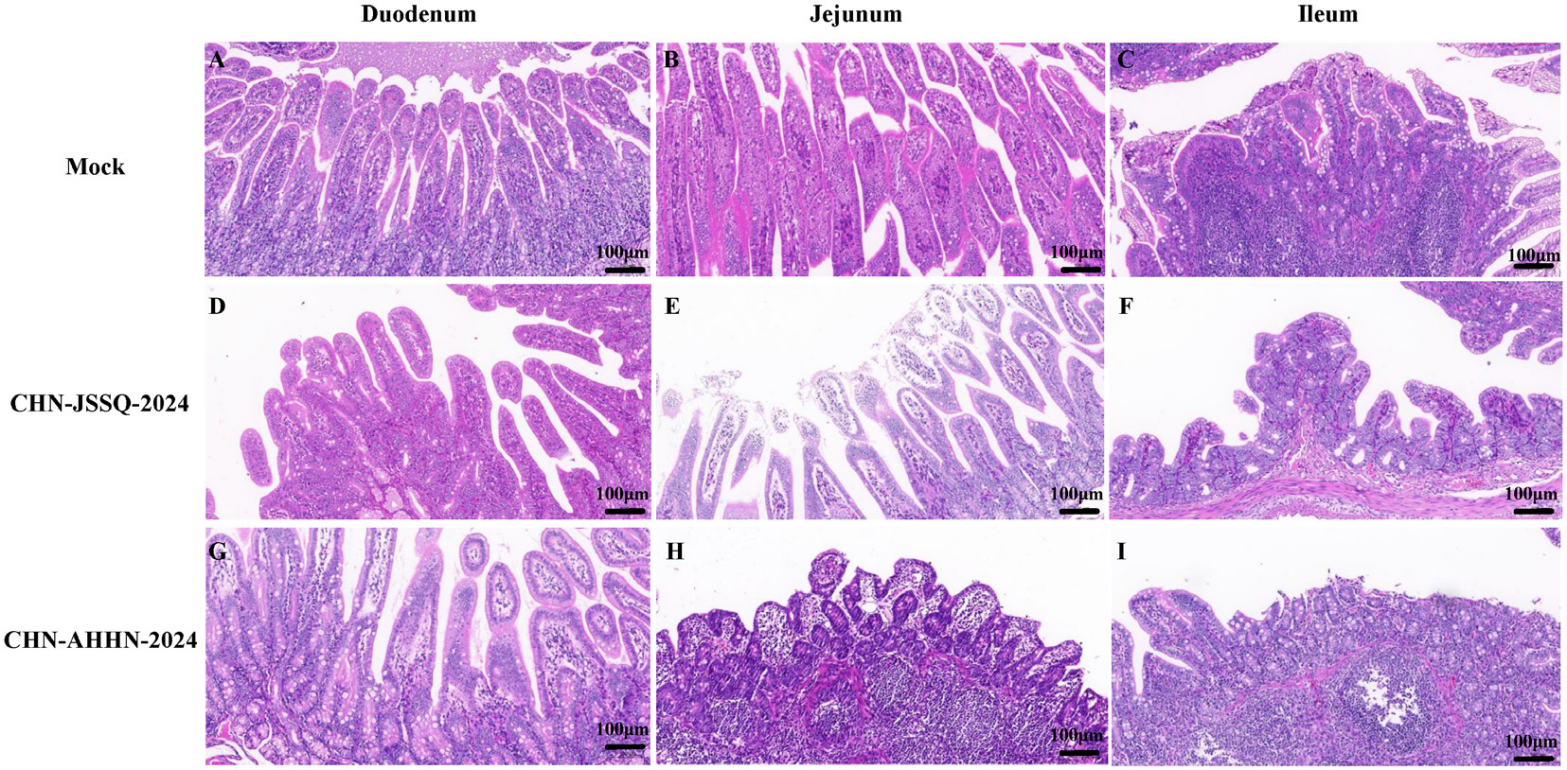
Illustrations of Intestinal Tract Gross Lesions Identified in Histopathological Analyses. Histopathological Sections of the Duodenum **(A)**, Jejunum **(B)**, and Ileum **(C)** in the Control Group. Histopathological Sections of the Duodenum **(D)**, Jejunum **(E)**, and Ileum **(F)** in Piglets Infected with CHN-JSSQ-2024. Histopathological Sections of the Duodenum **(G)**, Jejunum **(H)**, and Ileum **(I)** in Piglets Infected with CHN-AHHN-2024.

**Figure 7:**
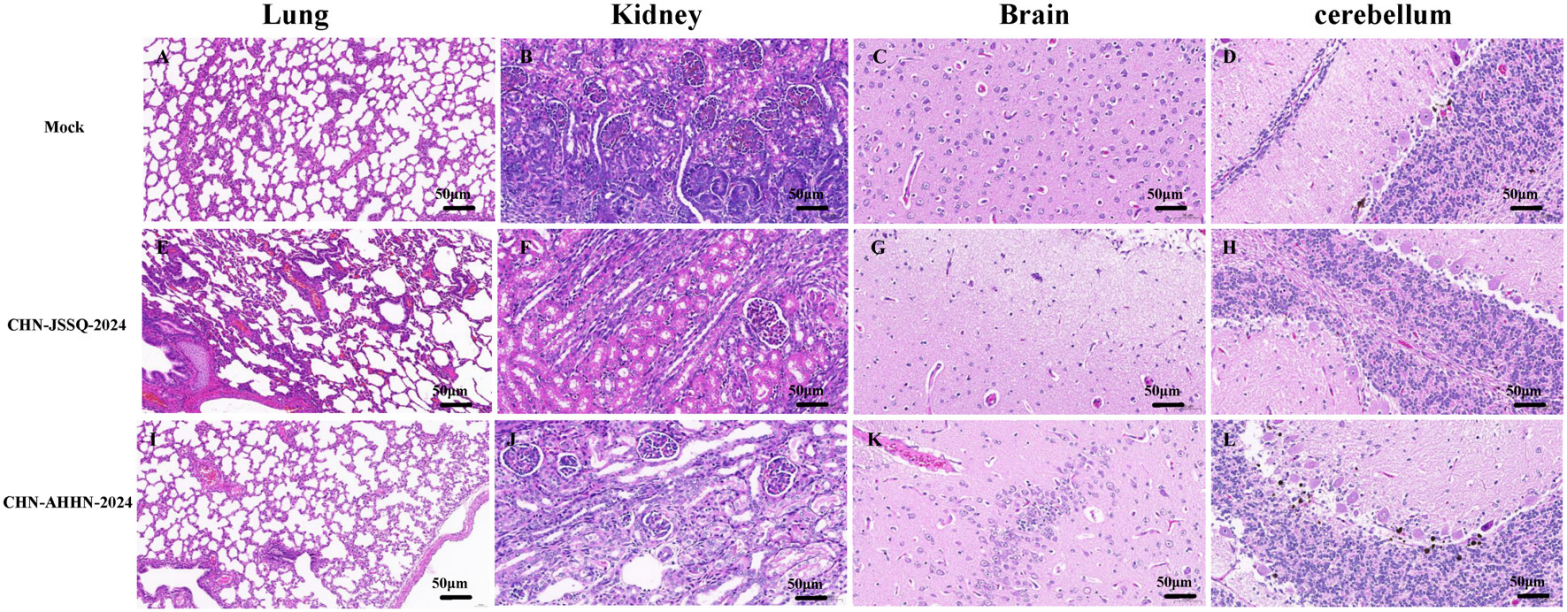
Illustrations of Organ Gross Lesions Identified in Histopathological Analyses. Histopathological Sections of the Lungs **(A)**, Kidneys **(B)**, Brain **(C)**, and Cerebellum **(D)** in the Control Group. Histopathological Sections of the Lungs **(E)**, Kidneys **(F)**, Brain **(G)**, and Cerebellum **(H)** in Piglets Infected with CHN-JSSQ-2024. Histopathological Sections of the Lungs **(I)**, Kidneys **(J)**, Brain **(K)**, and Cerebellum **(L)** in Piglets Infected with CHN-AHHN-2024.

To detect histological changes in piglets infected with the PDCoV CHN-ANHZ-2024 and CHN-JSSQ-2024 strains, necropsies were conducted on all piglets on day 3. In the MOCK group, the villi structures of the duodenum, jejunum, and ileum were intact and well-developed (**Figures 6A–C**). In the CHN-JSSQ-2024 group, the duodenum remained structurally intact, exhibiting slight hemorrhaging in the lamina propria and submucosa without any inflammatory response; the lamina propria of the jejunal villi displayed severe edema, while the ileal villi were well-developed, with no abnormal pathological changes noted (**Figures 6D–F**). In the CHN-AHHN-2024 group, the lamina propria of the duodenal villi exhibited edema, accompanied by mild dilation of the central lacteal; the jejunal villi demonstrated severe underdevelopment, and significant lymphocyte loss was noted in the ileal submucosa (**Figures 6G–I**). The lungs of the MOCK, CHN-JSSQ-2024, and CHN-AHHN-2024 groups exhibited a normal morphological structure without abnormal pathological changes (**Figures 7A, E, I**). The kidney tissues of the MOCK and CHN-JSSQ-2024 groups exhibited no abnormal pathological changes (**Figures 7B, F**). However, in the CHN-AHHN-2024 group, mild fibrosis was noted in the renal corpuscles, accompanied by degeneration and necrosis of renal tubular epithelial cells, along with the presence of hyaline casts (**Figure 7J**). The brain tissues of the MOCK and CHN-JSSQ-2024 groups appeared normal without any abnormal pathological changes (**Figures 7C, D, G, H**). In the CHN-AHHN-2024 group, inflammatory cell infiltration around the blood vessels in the cerebral cortex and the formation of small glial nodules, accompanied by a slight accumulation of glial cells, were noted (**Figures 7K, L**).

### PDCoV-CHN-ANHZ-2024 and CHN-JSSQ-2024 challenged piglets was detected by immunohistochemical analysis

**Figure 8:**
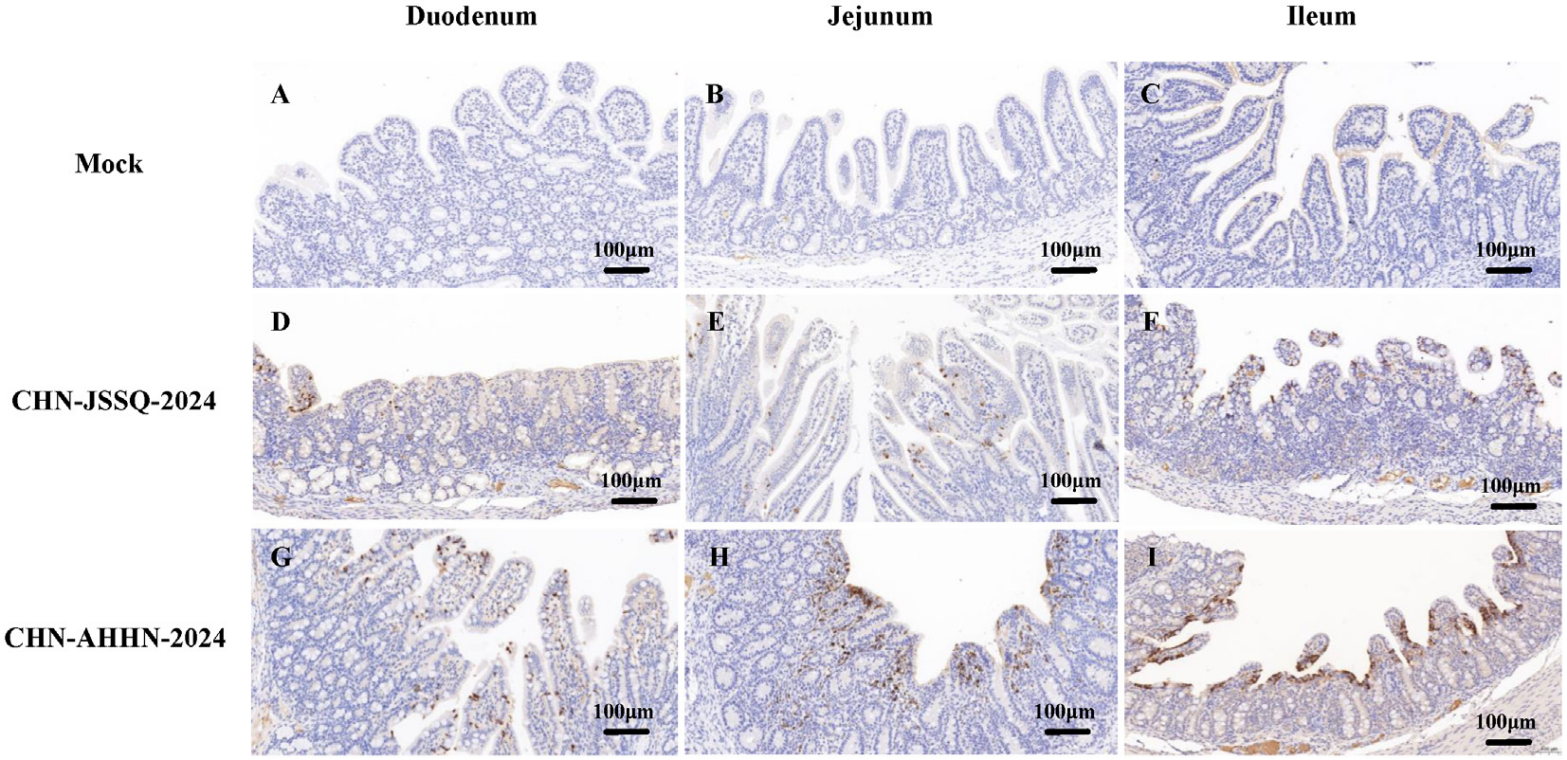
Illustrations of Intestinal Tract Gross Lesions Identified in Immunohistochemistry Analyses. Immunohistochemistry Analyses of the Duodenum **(A)**, Jejunum **(B)**, and Ileum **(C)** in the Control Group. Immunohistochemistry Analyses of the Duodenum **(D)**, Jejunum **(E)**, and Ileum **(F)** in Piglets Infected with CHN-JSSQ-2024. Immunohistochemistry Analyses of the Duodenum **(G)**, Jejunum **(H)**, and Ileum **(I)** in Piglets Infected with CHN-AHHN-2024.

**Figure 9:**
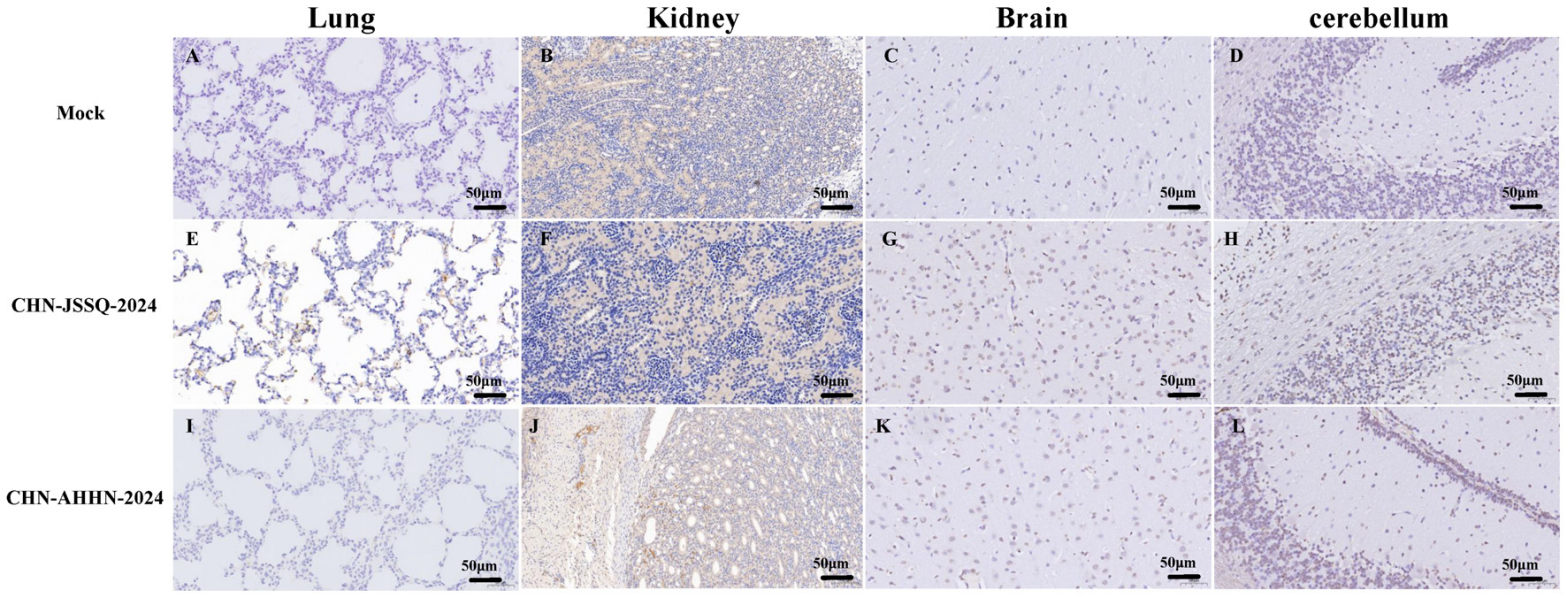
Illustrations of Organ Gross Lesions Identified in Immunohistochemistry Analyses. Immunohistochemistry Analyses of the Lungs **(A)**, Kidneys **(B)**, Brain **(C)**, and Cerebellum **(D)** in the Control Group. Immunohistochemistry Analyses of the Lungs **(E)**, Kidneys **(F)**, Brain **(G)**, and Cerebellum **(H)** in Piglets Infected with CHN-JSSQ-2024. Immunohistochemistry Analyses of the Lungs **(I)**, Kidneys **(J)**, Brain **(K)**, and Cerebellum **(L)** in Piglets Infected with CHN-AHHN-2024.

No positive signals were observed in the duodenum, jejunum, and ileum of the mock group (**Figures 8A–C**). Positive signals were detected in the epithelial cells of the duodenal, jejunal, and ileal villi in the CHN-JSSQ-2024 group(**Figures 8D–F**). A substantial number of positive signals were noted in the epithelial cells, lamina propria, and submucosa of the duodenum, jejunum, and ileum in the CHN-AHHN-2024 group(**Figures 8G–I**). In the CHN-JSSQ-2024 group, positive signals were primarily localized in the alveolar epithelial cells of the lungs (**Figure 9E**). No positive signals were detected in the kidneys (**Figure 9F**), brain (**Figure 9G**), and cerebellum (**Figure 9H**). In the CHN-ANHZ-2024 group, positive signals were detected in the renal interstitial cells (**Figure 9J**), while no positive signals were observed in the lungs (**Figure 9I**), brain (**Figure 9K**) and cerebellum (**Figure 9L**). In the mock group, no positive signals were detected in the lungs (**Figure 9A**), kidneys (**Figure 9B**), brain (**Figure 9C**), and cerebellum (**Figure 9D**).

### Viral infection by RNA scope

**Figure 10:**
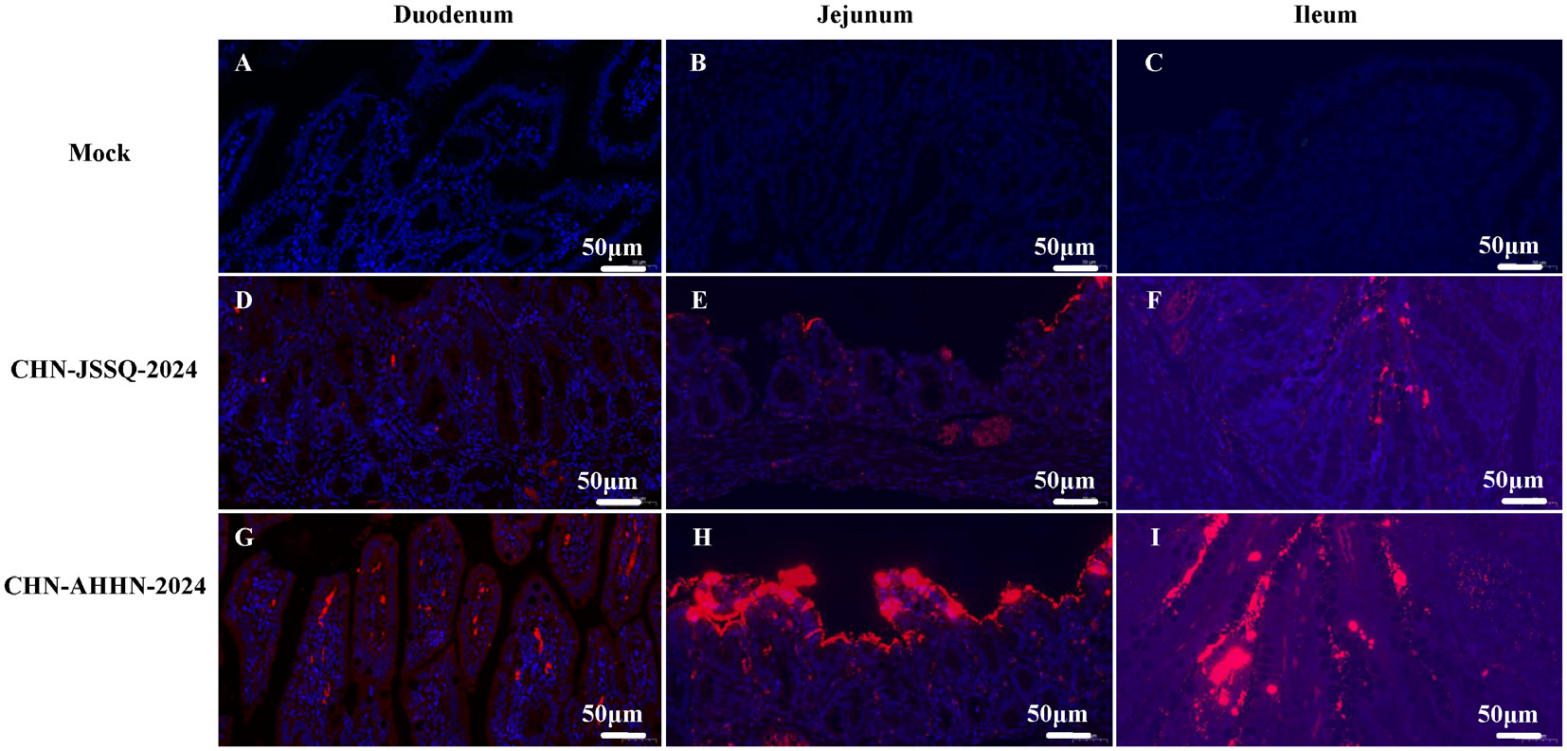
Viral RNA Distribution by ISH in the Intestinal Tract. PDCoV RNA was detected by RNA *in situ* hybridization in the Intestinal Tract of experimentally infected 3-day-old piglets euthanatized at 3 days post-inoculation and mock-infected piglets. Tissues were stained with a PDCoV-N target probe (red). Cell nuclei were counterstained with DAPI (blue). Viral RNA Distribution by ISH in the Duodenum **(A)**, Jejunum **(B)**, and Ileum **(C)** of the Control Group. Viral RNA Distribution by ISH in the Duodenum **(D)**, Jejunum **(E)**, and Ileum **(F)** of Piglets Infected with CHN-JSSQ-2024. Viral RNA Distribution by ISH in the Duodenum **(G)**, Jejunum **(H)**, and Ileum **(I)** of Piglets Infected with CHN-AHHN-2024.

**Figure 11:**
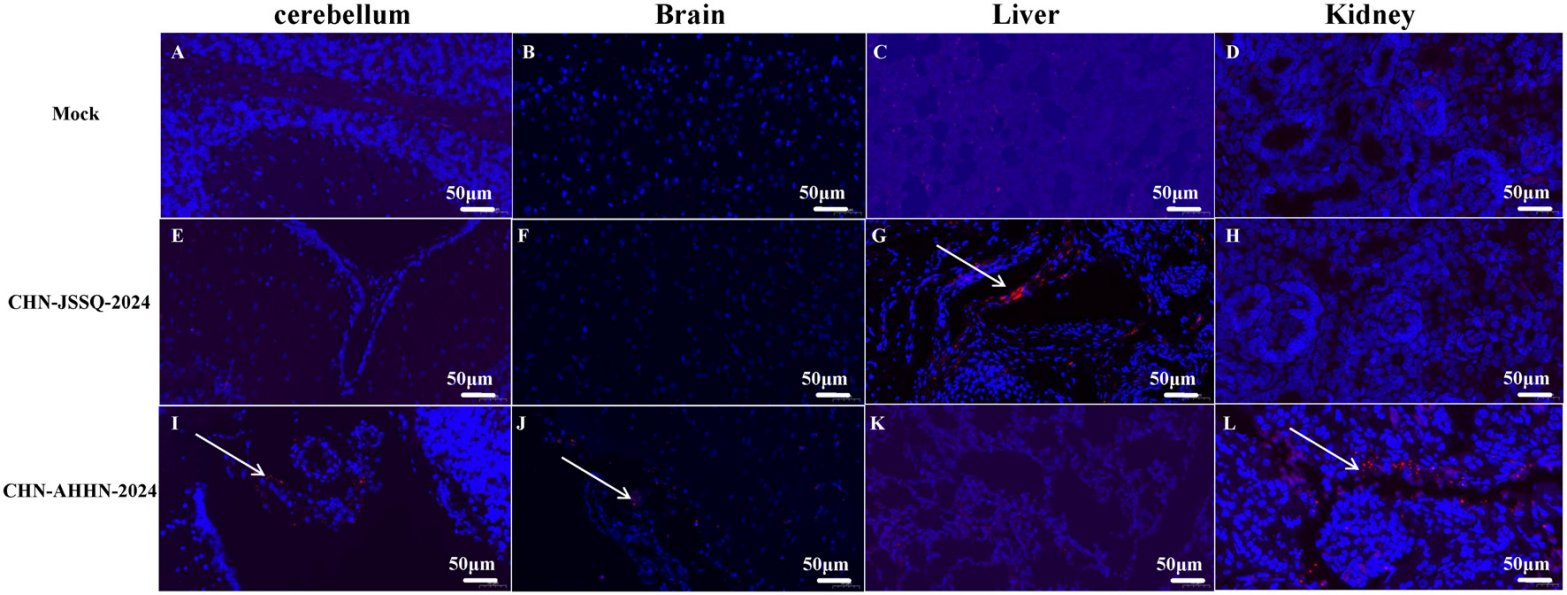
Viral RNA Distribution by ISH in Organs. PDCoV RNA was detected by RNA *in situ* hybridization in the organs of experimentally infected 3-day-old piglets euthanatized at 3 days post-inoculation and mock-infected piglets. Tissues were stained with a PDCoV-N target probe (red). Cell nuclei were counterstained with DAPI (blue). Viral RNA Distribution by ISH in the Lungs **(A)**, Kidneys **(B)**, Brain **(C)**, and Cerebellum **(D)** of the Control Group. Viral RNA Distribution by ISH in the Lungs **(E)**, Kidneys **(F)**, Brain **(G)**, and Cerebellum **(H)** of Piglets Infected with CHN-JSSQ-2024. Viral RNA Distribution by ISH in the Lungs **(I)**, Kidneys **(J)**, Brain **(K)**, and Cerebellum **(L)** of Piglets Infected with CHN-AHHN-2024. The white arrows indicate the locations of viral RNA Distribution.

RNA scope, a new technology for *in situ* RNA hybridization, was used to detect PDCoV nucleic acids *in situ* in piglet. Positive signals were detected in the villi of the duodenum, jejunum, and ileum of piglets infected with PDCoV-CHN-AHHN-2024 (**Figures 10G–I**) and CHN-JSSQ-2024 (**Figures 10D–F**). No positive signals were observed in the intestines of the mock group (**Figures 10A–C**). Viral RNA was detected in the brains, cerebella, and kidneys of piglets infected with the CHN-ANHZ-2024 strain (**Figures 11I, J, L**). In piglets infected with the CHN-JSSQ-2024 strain, viral RNA was detected in the lungs (**Figure 11G**). No viral RNA was detected in the control group and the untested positive organs (**Figures 11A–F, H, K**). In summary, PDCoV-CHN-ANHZ-2024 and CHN-JSSQ-2024 can cause intestinal lesions in three-day-old piglets. Viral RNA from PDCoV-CHN-ANHZ-2024 was primarily detected in the brain and kidneys, whereas PDCoV-CHN-JSSQ-2024 was primarily localized in the lungs.

## Discussion

PDCoV is a significant pathogen that affects pig herd health, resulting in substantial economic losses worldwide (More-Bayona et al., 2022). Various regions in China have reported cases of PDCoV infection in pig populations (Dong et al., 2015; Song et al., 2015; Liu et al., 2018). The ability to identify suitable receptors and efficiently enter host cells is a key factor in the interspecies transmission of viruses (Schrauwen and Fouchier, 2014). Numerous studies on coronaviruses have demonstrated that variations in the spike (S) protein can influence cross-species transmission and the emergence of coronaviruses in novel host populations (Perlman and Netland, 2009; Lau et al., 2013). In PDCoV, these functions are performed by the spike (S) protein, consisting of an S1 subunit that binds to the receptor and an S2 subunit that facilitates the fusion of the virus with the cell membrane (Xiong et al., 2018). The spike (S) protein exhibits high mutation rates and can readily undergo recombination and deletion events that result in altered tissue tropism, transmission routes, and host specificity (Ma et al., 2015).

In this study, two strains of PDCoV were isolated from the intestinal tissues of PDCoV-positive piglets, designated PDCoV-CHN-ANHZ-2024 and CHN-JSSQ-2024, both belonging to the Chinese lineage. Compared to other strains in the Chinese lineage, PDCoV-CHN-ANHZ-2024 and CHN-JSSQ-2024 exhibit amino acid mutations in the S1 subunit, which may result in distinct biological characteristics of the virus when infecting hosts. RDP4 recombination analysis indicates that CHN-JSSQ-2024 is a recombinant strain derived from DH1 and CHN-AHHN-2024, with the recombinant region located in the S2 subunit. Pathogenicity analysis revealed that PDCoV-CHN-AHHN-2024 has an 80% mortality rate in piglets, whereas CHN-JSSQ-2024 causes only mild diarrhea, which may be related to mutations in the S1 subunit. The spike protein’s S1 subunit plays a critical role in host receptor binding, and mutations in this region can directly impact viral entry into host cells (Li et al., 2017). In other coronaviruses, such as SARS-CoV and SARS-CoV-2, mutations in the spike protein have been linked to increased binding affinity to host receptors, facilitating more efficient infection (Hörnich et al., 2021). It is possible that the mutations identified in the S1 region of these PDCoV strains could enhance their ability to bind to porcine receptors, such as aminopeptidase N, or even enable binding to receptors in other species. This warrants further investigation, particularly in light of the zoonotic potential of coronaviruses.

Additionally, the observed mutations in the S1 subunit may indicate a heightened risk of cross-species transmission. In the case of other coronaviruses, such as MERS-CoV, mutations in the spike protein have been critical in enabling the virus to jump from camels to humans (Andersen et al., 2020). While PDCoV has primarily been studied in pigs, the possibility of transmission to other species, including humans, should not be overlooked. Experimental studies assessing receptor binding across various species would be valuable in understanding the full zoonotic potential of these PDCoV strains.

PDCoV exhibits a broad host range and infectivity, with positive detections reported in Asian leopard cats and Chinese ferret badgers (Dong et al., 2007). Additionally, it can infect cattle, mice, and poultry, and has even been detected in plasma samples from human children, demonstrating its extensive host range. The interspecies transmission of PDCoV and the associated risk of zoonotic diseases pose significant challenges to biosafety prevention efforts. Therefore, it is essential to obtain a deeper understanding of PDCoV infection dynamics and epidemiological features to develop more effective control measures.

In cases of PDCoV infection in neonatal piglets, it has been observed that, in addition to the intestines, other organs such as the heart, liver, spleen, lungs, and kidneys can also be infected (Hu et al., 2015; Ma et al., 2015). However, some reports on specific PDCoV strains infecting piglets have identified the virus solely in the intestines, with no detection in other organs (Chen et al., 2015; Jung et al., 2015; Hu et al., 2016). This research revealed that both strains of the virus can infect the intestines of piglets. CHN-AHHN-2024 predominantly affects the brain and kidneys, resulting in pathological damage, whereas CHN-JSSQ-2024 primarily targets the lungs. Previous studies have reported that PDCoV can induce mild interstitial pneumonia in the lungs (Ma et al., 2015); however, no pathological damage was observed in the lungs infected with CHN-JSSQ-2024, which may be attributed to an insufficient infection duration to cause damage.

Utilizing the novel technique RNA scope *in situ* hybridization (ISH), we detected viral RNA in infected organs, including the brain, cerebellum, kidneys, and lungs. Observing clear positive signals has been challenging in previous immunohistochemical studies of PDCoV-infected organs. The successful identification of viral RNA in the infected organs by ISH provides definitive evidence of PDCoV infection and establishes a foundation for studying the tissue tropism of PDCoV infections (Diao et al., 2021). SARS-CoV-2 primarily infects the lungs and bronchi but has also been detected in the kidneys, intestines, and brain, resulting in pathological damage (Scaldaferri et al., 2020; Diao et al., 2021; Wood et al., 2024). It can also infect other animals, including cats and dogs (Shi et al., 2020), exhibiting a wide host range and infectivity similar to PDCoV. Therefore, it is essential to gain a deeper understanding of the biological characteristics of PDCoV strains to prevent recombination or mutations that may increase the risk of cross-species transmission.

Limitation: The duration of the infection study in this research was relatively short. Viral RNA was detected in the lungs of piglets infected with strain A, but no significant pathological damage was found in the lungs, possibly due to the short infection time. No viral infection was found in organs such as the uninfected spleen and liver, and longer observation may be needed to assess the infection in these organs after viral infection. In addition, only two PDCoV strains were isolated in this study, and more PDCoV strains need to be isolated to study the infection characteristics of PDCoV.

In summary, this study isolated two strains of PDCoV exhibiting distinct pathogenicity and infection tropism. Phylogenetic analysis indicates that these strains belong to the Chinese lineage and exhibit variations in the S1 amino acids. The pathogenicity studies augment our understanding of the biological characteristics of PDCoV strains in China, providing valuable insights for the prevention of PDCoV in the country.

## Data Availability

The datasets presented in this study can be found in online repositories. The names of the repository/repositories and accession number(s) can be found in the article/supplementary material.
